# Sample manipulation and data assembly for robust microcrystal synchrotron crystallography

**DOI:** 10.1107/S2052252518005389

**Published:** 2018-04-19

**Authors:** Gongrui Guo, Martin R. Fuchs, Wuxian Shi, John Skinner, Evanna Berman, Craig M. Ogata, Wayne A. Hendrickson, Sean McSweeney, Qun Liu

**Affiliations:** a Photon Science Directorate, NSLS-II, Brookhaven National Laboratory, Upton, NY 11973, USA; b Biology Department, Brookhaven National Laboratory, Upton, NY 11973, USA; c GM/CA@APS, X-ray Science Division, Advanced Photon Source, Argonne National Laboratory, Argonne, IL 60439, USA; d Department of Biochemistry and Molecular Biophysics, Columbia University, New York, NY 10032, USA; e Department of Physiology and Cellular Biophysics, Columbia University, New York, NY 10032, USA

**Keywords:** microcrystals, microdiffraction, radiation damage, data analysis, multiple crystals, X-ray crystallography, structural biology

## Abstract

With the recent developments in microcrystal handling, synchrotron microdiffraction beamline instrumentation and data analysis, microcrystal crystallo­graphy with crystal sizes of less than 10 µm is appealing at synchrotrons. However, challenges remain in sample manipulation and data assembly for robust microcrystal synchrotron crystallography. Here, the development of micro-sized polyimide well-mounts for the manipulation of microcrystals of a few micrometres in size and the implementation of a robust data-analysis method for the assembly of rotational microdiffraction data sets from many microcrystals are described. The method demonstrates that microcrystals may be routinely utilized for the acquisition and assembly of complete data sets from synchrotron microdiffraction beamlines.

## Abstract 2

Micro-sized polyimide well-mounts for the manipulation of microcrystals and a data-assembly method for rotation data sets from many microcrystals are described.

## 1. Introduction   

X-ray crystallography is the predominant method of obtaining atomic-resolution structures for biomolecular investigation. Traditional macromolecular crystallography relies on the availability of large crystals that are often optimized from microcrystals for successful structural analysis. Solving structures directly from microcrystals of less than 10 µm is a very attractive prospect as it eliminates the need for optimization, which may be very challenging if not impossible. With the recent developments at synchrotron microdiffraction beamlines (Yamamoto *et al.*, 2017 ▸; Smith *et al.*, 2012 ▸), microcrystal synchrotron crystallography (MSX) became possible on microdiffraction beamlines (Stellato *et al.*, 2014 ▸; Gati *et al.*, 2014 ▸; Martin-Garcia *et al.*, 2017 ▸; Nogly *et al.*, 2015 ▸; Meents *et al.*, 2017 ▸; Beyerlein *et al.*, 2017 ▸; Diederichs & Wang, 2017 ▸; Coquelle *et al.*, 2015 ▸; Botha *et al.*, 2015 ▸; Ji *et al.*, 2010 ▸; Zeldin *et al.*, 2013 ▸). Serial femtosecond crystallography using X-ray free-electron lasers has accelerated the development of microcrystal crystallo­graphy (Chapman *et al.*, 2011 ▸; Boutet *et al.*, 2012 ▸; Spence, 2017 ▸; Schlichting, 2015 ▸), but only permits one still shot per microcrystal before its destruction by X-ray damage: the so-called ‘diffraction before destruction’ (Chapman *et al.*, 2011 ▸; Seibert *et al.*, 2011 ▸). As a comparison, with the reduced X-ray flux at synchrotron microdiffraction beamlines multiple exposures are possible from microcrystals through rotation data collection (Gati *et al.*, 2014 ▸; Roedig *et al.*, 2015 ▸; Rossmann *et al.*, 1979 ▸).

The intensity of the X-rays diffracted by a crystal is proportional to the volume of the crystal (Holton & Frankel, 2010 ▸). At a cryogenic temperature, the lifetime of cooled crystals may be extended more than 70 times (Garman & Weik, 2017 ▸; Owen *et al.*, 2006 ▸; Warkentin & Thorne, 2010 ▸). However, microcrystals may only survive a few degrees of data collection despite being cooled. To assist with MSX sample preparation and data collection at microdiffraction beamlines, workflows have been developed (Zander *et al.*, 2015 ▸; Bowler *et al.*, 2016 ▸; Coquelle *et al.*, 2015 ▸) and chips comprised of silicon nitride wafers or silicon matrix have also been developed (Coquelle *et al.*, 2015 ▸; Mueller *et al.*, 2015 ▸; Roedig *et al.*, 2015 ▸).

With the presence of radiation damage and heterogeneity in microcrystals, data assembly from incomplete rotation data sets is not trivial. How to effectively find the most compatible set of crystals and how to treat radiation damage remain the key issues for robust data assembly from microcrystals. We have previously proposed strategies for combining rotation data from multiple crystals (Liu *et al.*, 2012 ▸, 2013 ▸, 2014 ▸), and programs such as *BLEND* and *phenix.scale_merge* (Foadi *et al.*, 2013 ▸; Terwilliger *et al.*, 2016 ▸; Akey *et al.*, 2016 ▸; Zander *et al.*, 2016 ▸) have also been developed to handle data sets from multiple crystals. However, these existing data-assembly methods could not adequately address MSX data, where only a few degrees of radiation-damaged frames can be collected from a microcrystal.

Here, we present the development of polyimide micro-well-mounts for the harvesting and presentation of microcrystals for microdiffraction experiments. To treat radiation damage, we also developed a data-analysis method that allows the assembly of complete microdiffraction data sets from many microcrystals. Both methods address challenges in MSX and will pave the way towards routine and reliable MSX.

## 2. Methods   

### 2.1. Design and fabrication of micro-well-mounts   

**Figure 1:**
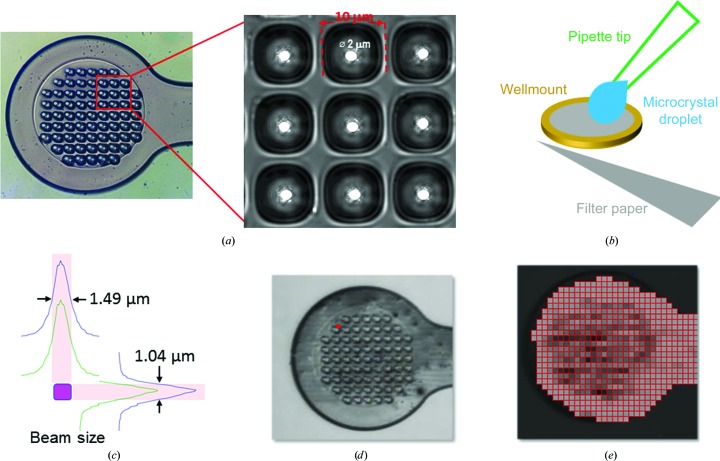
Manipulation of microcrystals for micro-crystallography. (*a*) Patterned well-mount. Inset: a high-resolution image of microwells to show their shape and dimensions. For better visualization, the 2 µm holes are highlighted by white circles. (*b*) A generic procedure for harvesting and cooling microcrystals. A micropipette is used to aspirate microcrystals and the microcrystal droplet is deposited onto the top side of the well-mount; solvent is then removed from the bottom side by using a filter paper and the well-mount is plunged into liquid nitrogen for cooling. (*c*) The 1.0 × 1.5 µm FMX beam profile measured at 12.6 keV. (*d*) A light microscopic view of a well-mount loaded with microcrystals in an orientation ready for raster scanning. (*e*) A raster-scan heat map from microcrystals on a well-mount.

We designed micro-sized well-mounts to manipulate microcrystals for microdiffraction experiments. These mounts feature the use of polyimide for a low X-ray scattering background, patterned micro-sized wells (10 µm width) for maintaining moisture and trapping microcrystals, and a 2 µm hole within a well for the removal of solvents using a filter paper (Fig. 1 ▸ *a*). The whole well-mount is about 250 µm in diameter with two different thicknesses: 3 µm for the film and 10 µm for the frame. These well-mounts were designed with MiTeGen MicroMesh M4 model No. 1 as a reference (custom manufactured by MiTeGen; http://www.mitegen.com) and were assembled onto 18 mm stainless-steel rods and SPINE caps for compatibility with standard cryocrystallography.

### 2.2. Microcrystal sample preparation   

Thaumatin protein (Sigma; catalog No. T7638) at a final concentration of 30 mg ml^−1^ was mixed with precipitant that consisted of 1.7 *M* tartrate, 100 m*M* ADA pH 6.5 in a ratio of 1:3 or 1:4(*v*:*v*). The mixed solution was sealed for nucleation and crystallization. After 3 h, the crystallization process was stopped by repeated centrifugation and buffer exchange with a stabilization solution consisting of 100 m*M* ADA pH 6.5, 0.9 *M* tartrate. Microcrystals were harvested by centrifugation followed by extrusion three times through an 8 µm Whatman filter. The dimensions of these crystals are about 1.5 × 3 µm as estimated by transmission electron microscopy.

Fig. 1 ▸(*b*) illustrates the procedure that we used to harvest and cool microcrystals. Instead of using the well-mount to scoop crystals from drops containing microcrystals, we used a micropipette to aspirate a drop of 0.5–1 µl of microcrystals. We then touched the microcrystal droplet on the top side of a well-mount, used a fine-tip filter paper to touch the bottom side of the well-mount to remove solvent, and plunged the well-mount into liquid nitrogen for rapid cooling.

### 2.3. Microdiffraction data collection and reduction   

We performed microdiffraction experiments on the Frontier Macromolecular Crystallographic Beamline (FMX) at National Synchrotron Light Source II (Fuchs *et al.*, 2016 ▸). The FMX beamline features a microbeam of 1 × 1.5 µm (FWHM; Fig. 1 ▸ *c*) focused by a KB mirror pair, an EIGER 16M hybrid pixel-array detector and a fast-scanning goniometer with a submicrometre sphere of confusion. The beam profile was determined at 12.6 keV by scanning a 30 nm chromium nanowire through the beam and measuring the emitted X-ray fluorescence signal. To align a well-mount, we centered the entire mount using a side view and then rotated the mount by 90° so that the surface of the well-mount was perpendicular to the X-ray beam (Fig. 1 ▸ *d*).

We used raster-scanning tools in the beamline data-collection program (*LSDC*) to find ‘hot-spot’ positions for diffraction data collection. We used a step size of 10 µm for raster scanning, and the returned ‘hot spots’ were mapped onto the raster grids to assist visualization and queue data collection (Fig. 1 ▸ *e*). We selected all ‘hot spots’ that demonstrated diffraction beyond 4.0 Å resolution, saved their positions and queued them for automated data collection. To record anomalous diffraction from protein S atoms to help our analysis, we collected all data at a relatively low energy (*E* = 7.0 keV). The focused beam flux is about 1.2 × 10^11^ photons s^−1^ at 7.0 keV with 16% transmission. At each selected ‘hot-spot’ position we collected 50 or 100 frames using a rotation angle of either 0.1 or 0.2° with an exposure time of 0.02 s per frame for fine-slicing data collection (Casanas *et al.*, 2016 ▸). At a sample-to-detector distance of 190 mm, the resolution limit (*d*_min_) was about 2.5 Å at the detector edge, and we collected a total of 128 partial data sets, each from a single crystal ‘hot-spot’. These partial data sets were collected from six well-mounts with eight to 64 crystals per well-mount.

All single-crystal data sets were indexed and integrated independently using *DIALS* (Waterman *et al.*, 2016 ▸; Winter *et al.*, 2018 ▸) and scaled and merged by using the *CCP*4 programs *POINTLESS* and *AIMLESS* (Evans & Murshudov, 2013 ▸; Evans *et al.*, 2011 ▸; see §[Sec sec3]3 for detailed data analysis). The data statistics for an optimal data set merged from 97 (reduced from 128 by rejection) statistically compatible data sets are listed in Supplementary Table S1.

### 2.4. Structure refinement   

*Phenix.refine* (Echols *et al.*, 2014 ▸; Afonine *et al.*, 2012 ▸) was used for structure refinement against the merged data set with an initial model from PDB entry 5lh1 (Schubert *et al.*, 2016 ▸). Friedel mates were treated as two reflections in all refinements, and the resultant Fourier coefficients (ANO and PHIANO) were used for the calculation of Bijvoet-difference Fourier maps. *Coot* (Emsley *et al.*, 2010 ▸) was used to check the map quality as well as for model and solvent adjustment. The stereochemistry of the refined structures was validated with *PROCHECK* (Laskowski *et al.*, 1993 ▸) and *MolProbity* (Chen *et al.*, 2010 ▸) for quality assurance. The refinement statistics for the optimal merged data set are listed in Supplementary Table S1.

## 3. Results   

### 3.1. Overall data-assembly strategy   

**Figure 2:**
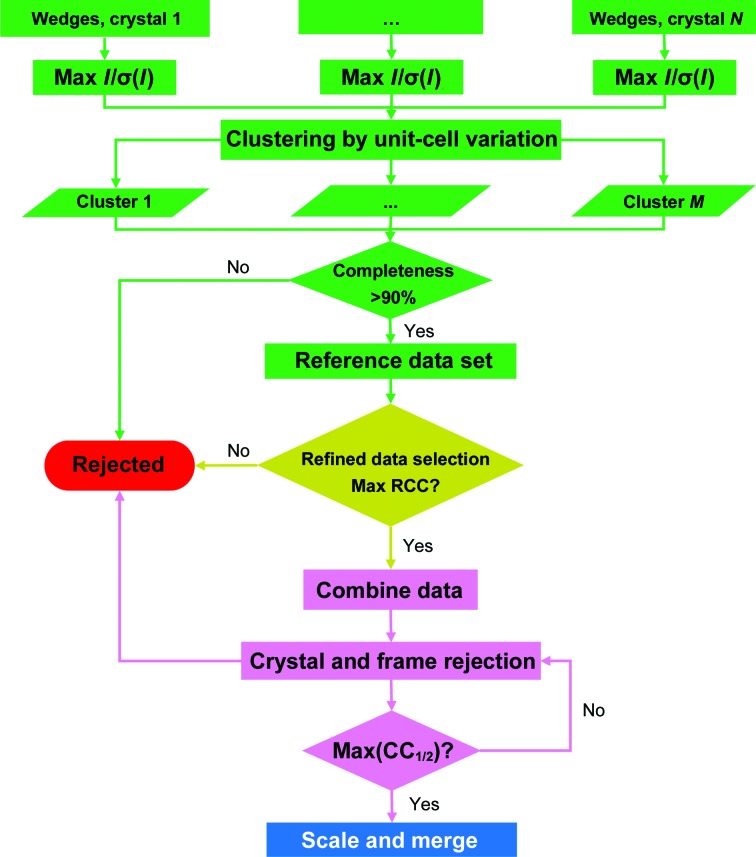
Strategy for data assembly. Firstly, the indexing and integration of single-crystal data sets are performed as cumulative wedges to find the maximum *I*/σ(*I*). Unit-cell variation analysis is used to obtain compatible crystals for a merged reference data set. Secondly, a refined selection of single-crystal data sets is based on the maximum RCC, where RCC is defined as the relative correlation coefficient of a single-crystal data set with the reference data set. Thirdly, iterative crystal and frame rejections are performed to obtain the final scaled and merged data for further analyses.

With crystals of linear dimensions of a few micrometres exposed to a focused micro-sized beam at a synchrotron, radiation damage is an intrinsic challenge. To attempt to overcome the radiation-damage problem, we devised a three-stage data-assembly strategy (Fig. 2 ▸). The first stage is to produce a complete reference data set from the set of single-crystal partial data sets. Each single-crystal data set is indexed and integrated as progressively cumulative data wedges. For each single-crystal data set, those wedges with highest *I*/σ(*I*) are selected. A clustering analysis based on unit-cell variation is then used to select a compatible set of crystals for merging into the reference data set. The second stage is to use the reference data set for a refined selection of crystals and frames within the single-crystal data sets, which we have performed based on the relative correlation coefficient (RCC) comparing data from a single-crystal data set with the reference data set. This yields a data set of ‘qualified’ frames from which finally merged data sets can be combined. The third stage seeks to produce candidate merged data sets from the ‘qualified’ frames, assuming that the data from a given cluster of crystals are sufficient: for example, that they have a merged completeness of greater than 90%. A process of iterative crystal rejection and frame rejection is used to generate a sorted succession of merged data sets at varied levels of stringency in rejection. These candidates are evaluated by measures of data quality and structural analysis.

### 3.2. Stage 1: production of the reference data set   

**Figure 3:**
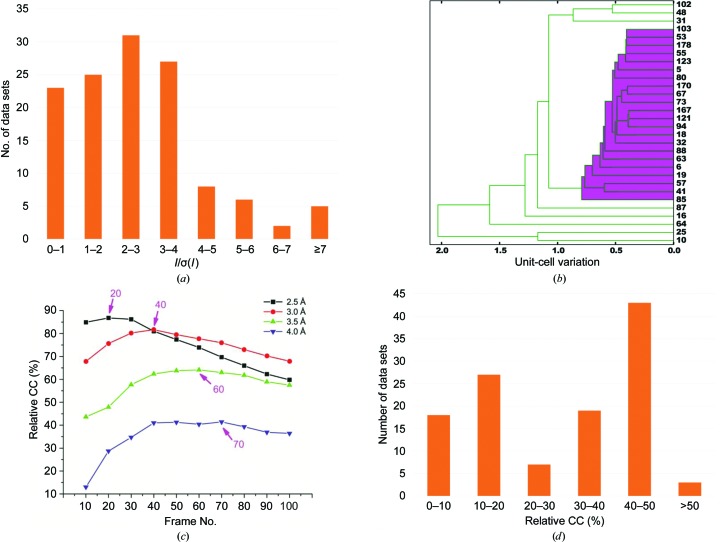
Data analysis of individual microcrystals. (*a*) Histogram distribution of *I*/σ(*I*) values for single-crystal data sets used to obtain the reference data set. (*b*) Unit-cell variation analysis for classification of single-crystal data sets in the reference data set. The eight crystals in the magenta-colored cluster are representative of the 96 crystals that co-clustered in the dendrogram. (*c*) RCC of a typical single-crystal data set to the reference data set at four different resolutions. (*d*) Histogram distribution of RCC values for the 117 selected single-crystal data sets.

We indexed and processed single-crystal data sets independently as progressively cumulative wedges. These microcrystals diffracted X-rays weakly, with an *I*/σ(*I*) of less than 3 for most single-crystal data sets (Fig. 3 ▸ *a*). Because *I*/σ(*I*) increases with multiplicity until radiation damage is overwhelming, we selected only cumulative wedges through the reaching of maximum *I*/σ(*I*). Using an *I*/σ(*I*) cutoff at 1.0, we selected 104 single-crystal data sets and performed unit-cell variation analysis to classify these data sets (Liu *et al.*, 2012 ▸; Foadi *et al.*, 2013 ▸; Giordano *et al.*, 2012 ▸). Our analysis showed that most microcrystals are clustered into a single cluster, with only eight outliers (Fig. 3 ▸ *b*). At this stage, the major cluster contained 96 crystals which clustered with representative crystals, shown in Fig. 3 ▸(*b*) as a magenta-colored branch of the dendrogram. The highest *I*/σ(*I*) frames from these selected crystals were then merged to obtain a reference data set. The reference data set used 1579 frames of the 9250 frames that were collected (17.1%).

### 3.3. Stage 2: refined selection of qualified data   

With a reference data set in hand, the frames from each partial single-crystal data set could be evaluated more reliably. We calculated the relative correlation coefficient (RCC) of the single-crystal data sets to the reference data set at four different resolutions (2.5, 3.0, 3.5 and 4.0 Å). For illustration, we display the progression of RCC for a representative data set with 100 frames in a 20° wedge (Fig. 3 ▸ *c*). The figure shows that the number of frames to reach the highest RCC is dependent on resolution, and is more susceptible to radiation damage at high resolution than at low resolution. For example, at 4.0 Å resolution up to 70 frames can be selected without compromising the RCC value, while at 2.5 Å resolution only 20 frames can be thus selected. We performed the RCC analysis for all wedged single-crystal data sets and, to include as many potentially useful data fames as possible, we used the maximum RCC at 4 Å as a cutoff for the selection of single-crystal cumulative wedges. That is, frames that yield a decreasing RCC at 4 Å for the cumulative wedge were rejected from further use (Fig. 3 ▸ *c*). With the criterion of RCC > 3%, we selected 126 single-crystal data sets. The goal here is to have an initial set of data for scaling and merging by *AIMLESS*. One can also use an RCC cutoff at a higher value or at a different resolution for initial crystal or frame selection. The clustering on unit-cell variations was slightly different using the refined selection here, and we now obtained 117 single-crystal data sets for the major cluster and used these for downstream analysis. The RCC histogram distribution of the selected single-crystal data sets is shown in Fig. 3 ▸(*d*). The complete data set at this stage included 3853 frames (41.7%).

### 3.4. Stage 3: combination into a sorted succession of merged data sets   

Our approach in this stage is to generate a succession of mergers sorted by the quality of data. We employ an iterative process of crystal and frame rejections before combining data into merged data sets, and we use the smoothed-frame *R*_merge_ (SmRmerge) as reported in *AIMLESS* (Evans & Murshudov, 2013 ▸) to judge the quality of each merger. We combined all 117 single-crystal data sets generated in stage 2 using *POINTLESS* and performed iterative data and frame rejection in *AIMLESS*. We first averaged the SmRmerge values for all frames within each single-crystal data set and then sorted the single-crystal data sets by their average SmRmerge values, 〈SmRmerge〉. The merger of all data qualified by the RCC tests in stage 2 for all 117 crystals was called merged data set 117, and iterative crystal rejection was begun by excluding the ten single-crystal data sets with the highest 〈SmRmerge〉 values. The remaining single-crystal data sets were recombined for another cycle of scaling and merging in *AIMLESS* to yield merged data set 107, from which updated 〈SmRmerge〉 values were calculated and used to exclude another ten crystals and initiate the next cycle of crystal rejection. In this manner, merged data sets 97, 87 *etc.* were generated and the rejection process was continued until the overall multiplicity was 5 or lower, which in this instance terminated with merged data set 17.

At each cycle of crystal rejection, we also performed frame rejections to remove radiation-damaged frames that might not contribute to the overall data quality. Owing to variations among crystals, there seems to be no uniform criterion on how many frames should be rejected per single-crystal data set. We therefore adopted a grid-search procedure and used SmRmerge for frame rejection. For each single-crystal data set, we first found the frame with the lowest SmRmerge, Min(SmRmerge), and we then defined the grid-rejection criterion as frame_rej = [Min(SmRmerge) × (1 + decay)] or none (effectively decay = ∞), where decay is the rejection ratio of 200, 150, 100, 50 or 10%. A lower decay means tighter rejection. Frames with SmRmerge values greater than frame_rej were rejected. Note that Min(SmRmerge) pertains to a particular single-crystal data set, not to the global minimum SmRmerge across all single-crystal data sets. Thereby, this process actually generated six merged data sets at each of 11 crystal-rejection levels to give a total of 66 merged data sets.

### 3.5. Assessment of data quality for merged data sets   

**Figure 4:**
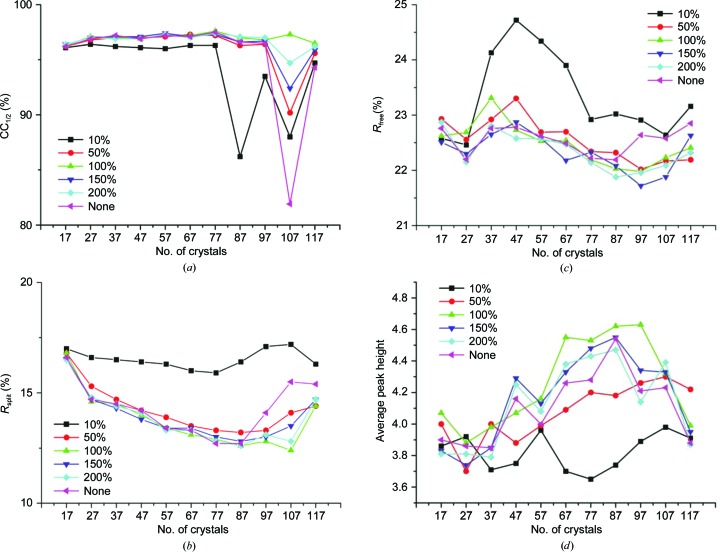
Analysis of merged data sets as a function of crystal and frame rejections. (*a*) CC_1/2_, (*b*) *R*_split_, (*c*) *R*_free_, (*d*) average Bijvoet-difference Fourier peak height. Within each plot, the curves correspond to a different extent of frame rejection after each cycle of crystal rejection. Frame rejection is shown at five different ratios, with 10% being the most stringent frame rejection and ‘None’ being no frame rejection.

We used CC_1/2_ and *R*_split_ as data-quality indicators to evaluate the effectiveness of the crystal- and frame-rejection process (Figs. 4 ▸ *a* and 4 ▸ *b*). Overall, our procedure is highly effective in identifying and rejecting poor single-crystal data sets and radiation-damaged frames. In our scheme, less compatible single-crystal data sets are identified by the establishment of large merged data-set numbers in the early stages of sorting and rejection. Including such single-crystal data sets caused unstable scaling in *AIMLESS*, as seen by fluctuations in CC_1/2_ and *R*_split_ with no frame rejections or overly stringent frame rejections (merged data sets 87–117). Moreover, *R*_split_ values tend to rise beyond merged data sets 87–97, indicating an adverse impact of including poor data, and they also increase when fewer crystals are used, probably because of the lower multiplicity. Frame rejection at 100% generally resulted in improved and smoothed CC_1/2_ and *R*_split_ values. On the other hand, the data quality was adversely affected by highly stringent rejection (10%), indicating that for microcrystals damaged frames are still contributing to the overall data quality.

### 3.6. Structure refinement and anomalous signal   

To further evaluate the effectiveness of our rejection criteria, we performed structure refinement of merged data sets using *phenix.refine *(Afonine *et al.*, 2012 ▸) and plotted the progression of *R*_free_ with respect to the number of crystals and the frame-rejection ratio (Fig. 4 ▸ *c*). The plot shows that although there are small glitches, data from more crystals tend to yield a lower *R*_free_, except for the last two merged data sets (107 and 117) where the merged data sets are less compatible and may have deteriorated the overall data quality. From our refinements, rejection ratios of between 100 and 200% appear to yield similar results independent of the number of single-crystal data sets included, suggesting that the frame-rejection strategy is quite effective and robust in excluding radiation-damaged frames.

Thaumatin contains one methionine residue and 16 cysteine residues that form eight disulfide bonds. We collected all of the single-crystal data sets at 7 keV, where the theoretical imaginary component of anomalous scattering (*f*′′) is 0.72 e for sulfur, and we estimate that the anomalous diffraction ratio from the protein is 2.2%. Consistently, our structure refinements show the presence of anomalous signal in merged data sets as judged by our *f*′′ refinement (Liu *et al.*, 2013 ▸). Taking merged data set 97 at a frame-rejection ratio of 100% as an example, the refined average *f*′′ for 17 sulfur sites is 0.75 e, which is close to the theoretical value of 0.72 e. We therefore calculated the Bijvoet-difference Fourier maps, used the *CCP*4 program *PEAKMAX* (Winn *et al.*, 2011 ▸) to find the six strongest peaks above 3σ and plotted the average peak height. Significant Bijvoet-difference Fourier peaks are found from most of the merged data sets, and among these merged data sets 67–97 (at multiplicities from 16 to 24) at a 100% rejection ratio all have average peak heights above 4.5σ (Fig. 4 ▸ *d*). This successful extraction of weak anomalous signals from merged data sets demonstrates that our strategy is robust and is relatively unaffected by crystal-rejection parameters. Thus, our method seems to be suitable for obtaining optimized data for refinement with the preservation of sensible anomalous signals.

## 4. Discussion   

### 4.1. Microcrystal manipulation   

The new well-mount that we have developed has proved to be effective for handling microcrystals. The use of polyimide for the mount greatly reduced background scattering and eliminated the need for silicon supports, which produce strong Bragg spots with significant absorption in low-energy experiments (Roedig *et al.*, 2016 ▸). From our experience, 10 µm wells and 2 µm holes are good for general use with microcrystals of a few micrometres and above. Therefore, this development provides an easy-to-implement method for the reliable harvesting, cooling and presentation of microcrystals for microdiffraction experiments. Although we used the well-mounts for just one type of thaumatin crystal which has the shape of a bipyramid, the design of the well-mounts and the rather straightforward sample-manipulation protocol are compatible with crystals of other lattices. We think that as long as crystals can be loaded onto a well-mount *via* a pipette they should attach and be trapped in various orientations by the well-mount during solvent removal from the bottom by a filter paper.

Without optimization of crystal slurry densities, our well-mounts have an inhomogeneous distribution of microcrystals: some may contain more than 60 well diffracting microcrystals, while some may contain only ten or fewer good microcrystals. The density of the crystal slurry may be increased by spinning down microcrystals and resuspending them in a smaller volume. Owing to variations in crystallization conditions and crystal morphologies, finding the optimal crystal density on a well-mount without stacking is a trial-and-error process. We imagine that the preparation of well-mounts with several concentrations of microcrystals followed by diffraction testing on a beamline would provide sufficient experimental data to allow the preparation of optimized experiments. *DIALS* can be used to detect multiple lattices using these sacrificial well-mounts. The current size of the well-mount is only 250 µm in diameter. To load more crystals onto one well-mount, a diameter of 1–2 mm may be useful (Coquelle *et al.*, 2015 ▸). When cooling microcrystals on the well-mount, we did not observe ice-ring diffraction even without adding any cryoprotectant. This is perhaps owing to our rather complete solvent removal. Nevertheless, cryoprotectants can be conveniently added either to crystallization drops or to the stabilization solution containing microcrystals.

### 4.2. Outlier data and frame rejection   

In comparison to large crystals, microcrystals contain fewer molecules and are more sensitive to manipulation and changing environment, as shown by changes in unit-cell parameters and diffraction intensities (Farley *et al.*, 2014 ▸). Therefore, to merge data from microcrystals, we need to detect these outliers effectively and reject them. With complete data from a single crystal, we have used unit-cell variation analysis and diffraction-dissimilarity analysis to ensure that only data from compatible crystals are combined together (Liu *et al.*, 2012 ▸; Giordano *et al.*, 2012 ▸). For the partial data typical for microcrystals, unit-cell variation analysis remains effective; however, the number of reflections in common between partial single-crystal data sets is typically insufficient for reliable diffraction-intensity correlation analysis. While it is possible to collect a medium-quality reference data set from a single crystal to assist microcrystal data assembly (Hanson *et al.*, 2012 ▸), the selection of single-crystal data sets might be biased by the reference if there are variations in unit-cell dimensions and diffraction intensity. Because we used *AIMLESS* for all scaling and merging work, for compatibility we also used *AIMLESS* to calculate 〈SmRmerge〉 for iterative sorting and outlier crystal rejection. In our analysis, we can reliably detect incompatible crystals and reject them effectively (Figs. 4 ▸ *a* and 4 ▸ *b*). Although we could obtain a complete merged data set with 17 crystals (a multiplicity of about 5), all quality indicators show that including more single-crystal data sets improves the data quality and enhances weak anomalous signals (Figs. 4 ▸ *a*–4*d*).

How do we know the point at which the extent of damage to the frames is too serious for these frames to be useful? Our grid-search frame rejection may provide a practical route towards the rational treatment of radiation damage. Here, we tested five different rejection ratios and we found that a value of between 100 and 200% gave a reasonable smoothness to the merged data sets, as well as a maximum CC_1/2_ and a minimum *R*_split_, independent of the number of single-crystal data sets that were included. We thus propose that for the rejection of radiation-damaged frames, one should try different rejection ratios and select those that give optimal CC_1/2_ and *R*_split_ values (Figs. 4 ▸ *a* and 4 ▸ *b*).

Compared with SFX data, a multiplicity of 5 for microcrystals is rather low. Here, we used a multiplicity of 5 to show that even at such a low multiplicity it is possible to assemble a complete data set with reasonable structure-refinement statistics (*R*_free_ = 0.23) from as few as 17 crystals. This suggests that it is possible to obtain single-crystal quality data from a limited number of microcrystals by collecting rotation data on a synchrotron microdiffraction beamline.

### 4.3. Weak diffraction signals   

Bijvoet-difference Fourier peaks at sulfur positions provide sensitive measures of data accuracy, and we have used such peaks to detect anomalous signals in these merged data sets (Fig. 4 ▸ *d*). Supplementary Fig. S1 shows Bijvoet-difference Fourier peaks for merged data sets between 17 and 117 with a frame-rejection ratio of 100%. Although there are four peaks in merged data set 17, including more single-crystal data sets enhanced the anomalous signal and more peaks appeared, with eight peaks (out of nine) clearly resolved in merged data set 97. However, consistent with measures of *R*_split_ (Fig. 4 ▸ *b*), the inclusion of all data (merged data set 117) is detrimental to the anomalous signal (Fig. 4 ▸ *d*); peaks 1, 3 and 6 in data set 97 almost disappear here (Supplementary Figs. S1*e* and S1*f*). Similarly, Supplementary Fig. S2 shows the Bijvoet-difference Fourier peaks at different rejection ratios for the merged data set 97. Clearly, a rejection ratio of 100% is consistent with the detection of eight peaks. However, using the merged data set 97 we were unable to find the sulfur substructure with either *SHELXD* (Sheldrick, 2010 ▸) or *phenix.hyss* (Zwart *et al.*, 2008 ▸). In an attempt to improve anomalous signals, we tested local scaling and anomalous signal optimization as implemented in *phenix.scale_merge* (Terwilliger *et al.*, 2016 ▸). However, the data after local scaling and anomalous signal optimization showed increased *R*_free_ and decreased Bijvoet-difference Fourier peak heights, suggesting that the anomalous signals are too weak for more reliable extraction. We then used the known substructure derived from the PDB for SAD phase calculation followed by density modification. We calculated the map correlation coefficients (mapCCs) between these SAD-phased maps and the model map, and found that the mapCCs are around 15%, suggesting that the signal, while present, is too weak for *de novo* SAD phasing from fewer than 100 microcrystals. Nevertheless, since our data-analysis procedure is robust, we propose that native SAD structure determination will be feasible with increased numbers of microcrystals and possible improvements in experimental conditions, such as a lower X-ray energy.

## 5. Concluding remarks   

Microcrystals of a few micrometres are challenging to handle. Subsequent microdiffraction data assembly in the presence of radiation damage is not straightforward to attain optimal results. Here, we used thaumatin microcrystals to demonstrate robust microcrystal handling with micro-sized well-mounts and data assembly from small-wedged rotation data sets. By progressively processing single-crystal data sets, by using a reference data set and by using iterative crystal and frame rejection, our data-assembly procedure is quite robust. By combining these strategies, our method provides an attractive route for optimized MSX experiments at synchrotrons. Beyond MSX, our data-assembly strategy may work equally well for microdiffraction from larger crystals.

## Supplementary Material

PDB reference: thaumatin structure from microcrystals, 6c5y

Supporting figures and table. DOI: 10.1107/S2052252518005389/it5015sup1.pdf
