# Supplementary material for: Sample manipulation and data assembly for robust microcrystal synchrotron crystallography
Source: IUCrJ. 2018 Apr 19;5(Pt 3):238–46. doi: 10.1107/S2052252518005389 (PMC5929371; doi:10.1107/S2052252518005389)
Supplement: Supplementary file 1 [file m-05-00238-sup1.pdf]

IUCrJ

Volume 5 (2018)

Supporting information for article:

Sample manipulation and data assembly for robust microcrystal synchrotron crystallography

Gongrui Guo, Martin R. Fuchs, Wuxian Shi, John Skinner, Evanna Berman, Craig M. Ogata, Wayne A. Hendrickson, Sean McSweeney and Qun Liu

Table S1 Data-collection and refinement statistics for merged data set 97 (100%) and reference data

**Data collection**	Merged dataset 97	Reference dataset
Beamline	FMX (NSLS-II)	FMX (NSLS-II)
Wavelength (Å)	1.76	1.76
Space group	$P4_12_12$	$P4_12_12$
Cell dimensions	57.58, 150.48	57.55, 150.54
Solvent content (%)	53.0	53.0
Bragg spacings (Å)	40-2.56 (2.71-2.56)	40-2.56 (2.71-2.56)
Total reflections	223965	136498
Unique reflections	9420	9420
Completeness (%)	100.0 (100.0)	99.9 (99.8)
I/σ(I)[1]	8.9 (4.1)	11.6 (6.2)
R_{split}	0.124 (0.287)	0.380 (0.404)
Multiplicity	23.8 (22.7)	14.5 (14.1)
$CC_{1/2}$ (%)	0.967 (0.873)	0.811 (0.761)
**Refinement**		
Resolution (Å)	2.56	
No. reflections	9354	
R_{work}/R_{free}	0.182/0.220	
No. atoms	1669	
Average B (Å2)	16.3	
R.m.s deviations		
Bond length (Å)	0.002	
Bond angle (°)	0.529	
PDB code	6C5Y	

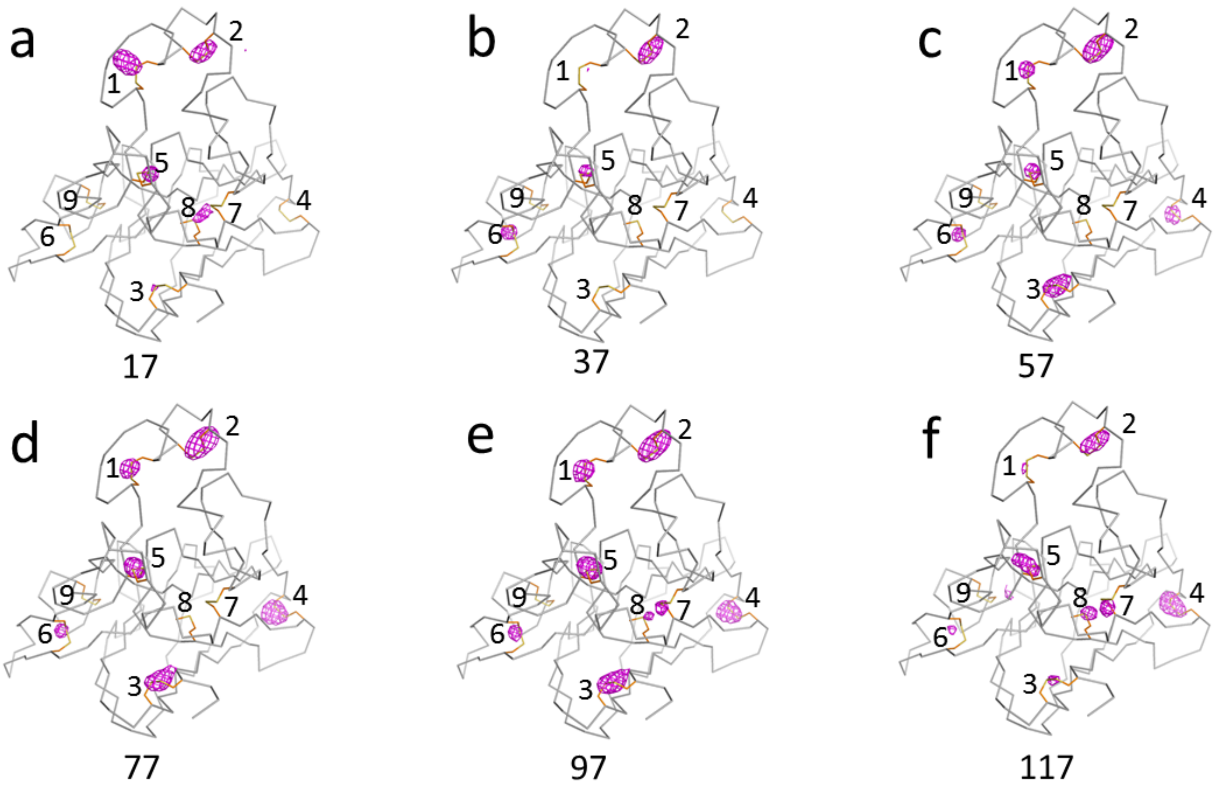

Figure S1 Bijvoet-difference Fourier peaks after crystal rejection. Bijvoet-difference Fourier peaks for anomalous scatterers (sulfur) were shown as magenta isomeshes and contoured at 3.0σ. The data shown here are from frame rejection ratio at 100%. The numbers and sticks indicate respectively, the positions and residues of anomalous scatters in the structure: 1: Cys149-Cys158; 2: Cys159-Cys164; 3: Cys9-Cys204; 4: Cys121-Cys193; 5: Cys134-Cys145; 6: Cys56-Cys66; 7: Cys126-Cys177; 8: Met 122; 9: Cys71-77.

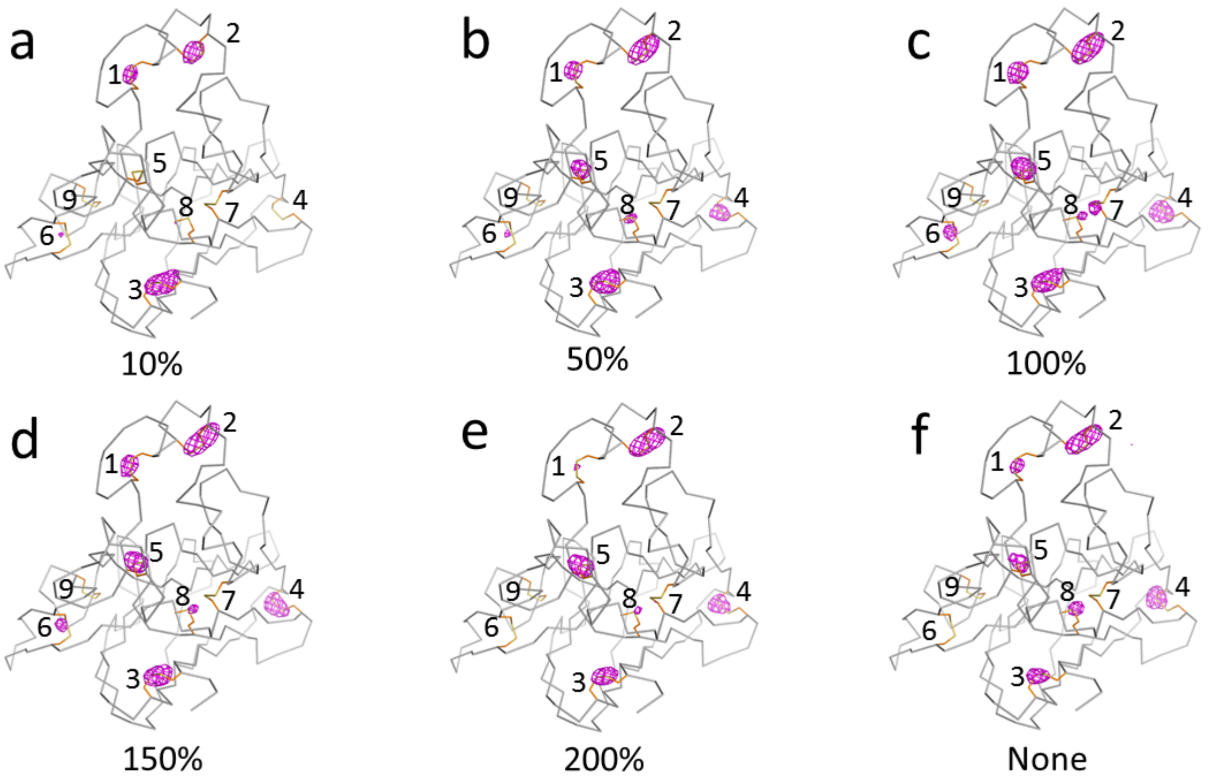

Figure S2 Bijvoet-difference Fourier peaks after frame rejection. Bijvoet-difference Fourier peaks for anomalous scatterers were shown as magenta isomeshes and contoured at 3.0σ. The data shown here are merged dataset 97 with various frame rejection ratios. The numbering is the same as S2.
